# Detection of Oil Chestnuts Infected by Blue Mold Using Near-Infrared Hyperspectral Imaging Combined with Artificial Neural Networks

**DOI:** 10.3390/s18061944

**Published:** 2018-06-15

**Authors:** Lei Feng, Susu Zhu, Fucheng Lin, Zhenzhu Su, Kangpei Yuan, Yiying Zhao, Yong He, Chu Zhang

**Affiliations:** 1 College of Biosystems Engineering and Food Science, Zhejiang University, Hangzhou 310058, China; lfeng@zju.edu.cn (L.F.); sszhu@zju.edu.cn (S.Z.); zhaoyy@zju.edu.cn (Y.Z.); yhe@zju.edu.cn (Y.H.); chuzh@zju.edu.cn (C.Z.); 2 Key Laboratory of Spectroscopy Sensing, Ministry of Agriculture, Hangzhou 310058, China; 3 State Key Laboratory for Rice Biology, Institute of Biotechnology, Zhejiang University, Hangzhou 310058, China; fuchenglin@zju.edu.cn; 4 College of Life Sciences, Zhejiang University, Hangzhou 310058, China; yuankp@zju.edu.cn

**Keywords:** chestnuts, hyperspectral imaging technology, blue mold, artificial neural networks

## Abstract

Mildew damage is a major reason for chestnut poor quality and yield loss. In this study, a near-infrared hyperspectral imaging system in the 874–1734 nm spectral range was applied to detect the mildew damage to chestnuts caused by blue mold. Principal component analysis (PCA) scored images were firstly employed to qualitatively and intuitively distinguish moldy chestnuts from healthy chestnuts. Spectral data were extracted from the hyperspectral images. A successive projections algorithm (SPA) was used to select 12 optimal wavelengths. Artificial neural networks, including back propagation neural network (BPNN), evolutionary neural network (ENN), extreme learning machine (ELM), general regression neural network (GRNN) and radial basis neural network (RBNN) were used to build models using the full spectra and optimal wavelengths to distinguish moldy chestnuts. BPNN and ENN models using full spectra and optimal wavelengths obtained satisfactory performances, with classification accuracies all surpassing 99%. The results indicate the potential for the rapid and non-destructive detection of moldy chestnuts by hyperspectral imaging, which would help to develop online detection system for healthy and blue mold infected chestnuts.

## 1. Introduction

Chestnuts, a Chinese specialty, are nutritious and contain plenty of starch, protein and fat. Chestnuts are widely planted in China and their production is increasing year by year [1]. However, chestnuts frequently suffer from serious dehydration, mildew infestation and pest injuries during improper storage and subsequent transportation [2,3,4].

Chestnut mildew is a major cause of chestnut loss [5,6]. The removal of moldy chestnuts from good ones mainly depends on manual selection or salt water floatation, which are inefficient [7]. At the early stage of spoilage, the mold size is too small to be distinguished by visual inspection. Besides, chestnuts may become moldy inside the shell, which makes selection even more difficult. These situations will result in high error rates during sorting, therefore, development of a rapid, accurate and non-destructive detection method for moldy chestnuts is of great significance for chestnut storage and processing.

Hyperspectral imaging combines imaging and spectroscopy technology. Within hyperspectral images, spectral information and image information can be simultaneously extracted from target objects [8,9,10]. Hyperspectral imaging can be used to visualize and manifest the internal and external attributes of the detection object, and has a series of advantages such as high spectral resolution and a more comprehensive information content [11,12,13,14]. The most commonly used way to obtain spectral information in hyperspectral images is to define a region of interest (ROI) for data extraction [10,11,12,13]. Hyperspectral imaging technology has been widely studied in agricultural and food industry [11,12]. Wu et al. developed a laboratory hyperspectral imaging system to capture hyperspectral scattering images of beef surfaces in order to predict and classify beef tenderness [15]. Lohumi et al. used Fourier transform near infrared (FT-NIR) spectroscopy to determine whether watermelon seeds were viable [16]. What’s more, hyperspectral imaging technology was also applied to detect mildew [13,14,15,16,17]. Shahin et al. assessed the mildew levels in wheat samples according to the spectral characteristic of bulk grains and partial least square regression models were established to predict mildew levels [17]. Zhang et al. detected powdery mildew of winter wheat with leaf-level hyperspectral measurements. They measured hyperspectral reflectance of leaves infected with powdery mildew and normal leaves. Two regression models were used to evaluate the effect and both models showed desirable results [18]. Knauer et al. used spatial-spectral analysis of hyperspectral images to improve classification accuracy of powdery mildew infection levels of wine grapes and they found the spatial spectral approach improved the detection of slight infection levels compared with pixel-wise spectral data analysis [19]. Shahin et al. did a comparison between visible near-infrared imaging and near-infrared spectra based on spectral characteristics of bulk samples of mildew damage in soft red winter wheat and the former method showed a better detection result [20]. Tian et al. found the accuracy rate reached nearly 90% using hyperspectral image technology to detect cucumber downy mildew [21].

Artificial neural networks (ANNs) are computing systems inspired by biological neural networks. ANNs accomplish tasks by analyzing examples and they are widely used where problems are difficult to be expressed in a traditional computation algorithm using rule-based programming. The basic units of ANNs are artificial neurons, analogous to the axons of the biological brain. Signals can be transmitted through the connection between neurons. The receiving neurons can process the signal and then transmit the signal to its downstream neurons. Generally, neurons are organized in layers, where different kinds of transformations of inputs are performed. Nowadays, ANNs have been applied to various fields, such as computer vision, machine translation, speech recognition, social network filtering and many other domains [22,23,24].

Analyzing the hyperspectral data with artificial neural networks is an effective way to build a classification model. Sun et al. built a set of lettuce leaf nitrogen level identification models based on the hyperspectral image technology and the extreme learning machine (ELM) pattern recognition method. The training time and classification accuracy rate of ELM model were 0.062304 s and 100%, respectively [25]. Zhou et al. compared the effects of six preprocessing methods on back propagation neural network (BPNN) models to identify moldy Chinese chestnuts. The results showed that the classification accuracies of qualified chestnuts, ostensibly moldy chestnuts and internally moldy chestnuts in the prediction set reached 94.74%, 94.44% and 92.31%, respectively [7].

This work aimed to explore the feasibility of using spectral information obtained from hyperspectral images combined with neural networks, including ELM, BPNN, evolutionary neural networks (ENN), general regression neural network (GRNN) and radial basis neural network (RBNN) models to detect oil chestnuts infected by blue mold.

## 2. Materials and Methods

### 2.1. Sample Preparation

The oil chestnut samples were collected from Anji (Zhejiang Province, China). Firstly, the chestnuts were cleaned and air-dried. Next, the samples were divided into two groups, healthy chestnuts and healthy chestnuts to be infected. To obtain enough infected chestnuts, some chestnuts infected by blue mold were placed among normal chestnuts under 85% humidity and 25 °C for one week. There were 264 healthy chestnuts and 264 infected chestnuts prepared for this study. Then, these chestnuts were randomly split into the calibration set and prediction set at the ratio of 2:1, with 176 healthy samples and 176 infected samples in the calibration set and 88 healthy samples and 88 infected samples in the prediction set. The entire sample set (264 healthy chestnuts and 264 infected chestnuts) was imaged in multiple batches (eight batches for healthy samples and 12 batches for moldy samples) because of the size limitations of mobile platform.

### 2.2. Hyperspectral Imaging System

**Figure 1:**
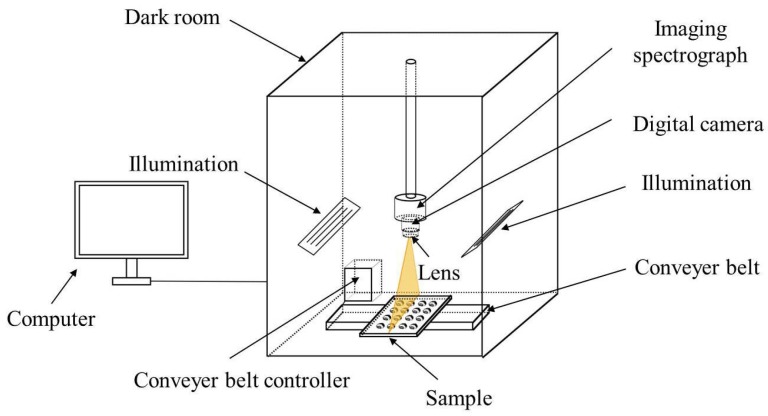
Hyperspectral imaging system.

The hyperspectral imaging system was provided by the Zhejiang University College of Biosystems Engineering and Food Science. The system was installed at a black sealed box. It could acquire 256 spectral image bands in the region of 874–1734 nm with spectral resolution of 5 nm. As shown in Figure 1, the system included an ImSpector V10E imaging spectrograph (Spectral Imaging Ltd., Oulu, Finland), a Xeva 922 digital camera (Xenics Infrared Solutions, Leuven, Belgium), an OLES22 lens (Spectral Imaging Ltd.), two 150 W tungsten halogen lamps (3900 Lightsource, Illumination Technologies Inc., Elbridge, NY, USA) placed symmetrically, a conveyer belt (Isuzu Optics Corp., Zhubei City, Taiwan). Images acquired by the system was analyzed by a data acquisition and processing software (Xenics N17E, Isuzu Optics Corp.). During image acquisition, the sample moves with the conveyer belt while the camera remains still. A complete hyperspectral image cube was obtained as the conveyor belt moved along its x-direction. The size of the hyperspectral image cube (cropped with the ENVI 4.6 hyperspectral image analysis software, ITT, Visual Information Solutions, Boulder, CO, USA) is 600 pixels vertical × 320 pixels horizontal × 256 spectral bands [26].

### 2.3. Hyperspectral Image Acquisition and Correction

A black plate with low reflectance was used as background in the study. With this black plate, chestnuts could easily be isolated from the background. During the image acquisition, the height between the camera and the platform was 26.5 cm, the moving speed of the platform was 23.5 mm/s, and the exposure time of the spectral camera was 2 ms.

In this study, the accuracy of reflectance would be influenced by the illumination source and the sensitivity of detector. The original image (*I_raw_*) was calibrated by two reference standards. Black reference image (*I_dark_*) was obtained by covering the lens with lens cap whose reflectivity was about 0%. A pure white Teflon board which had a high reflectivity (about 100%) aligned with the lens to obtain a white reference image (*I_white_*). The calibrated image (*I_c_*) could be calculated as follow [27]:

(1) $$I_{c}=\frac{I_{raw}-I_{dark}}{I_{white}-I_{dark}}$$

### 2.4. Hyperspectral Image Preprocessing and Spectral Data Extraction

**Figure 2:**
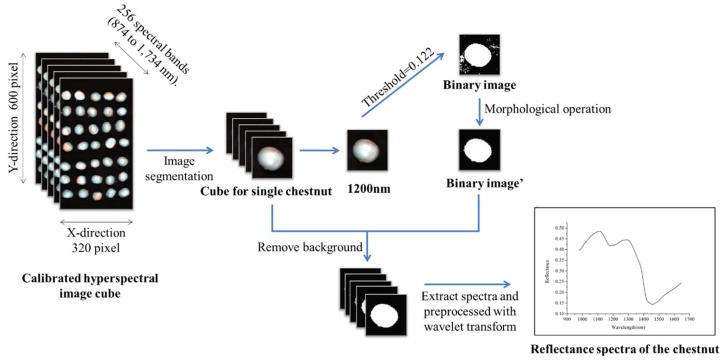
Procedures of hyperspectral image preprocessing and spectral data extraction.

In this study hyperspectral image data were acquired in the 874–1734 nm spectral region. Figure 2 shows the hyperspectral image preprocessing and spectral data extraction procedures [26]. After image acquisition, a single chestnut was isolated from the original hyperspectral cube using ENVI 4.6. To extract spectral data from the hyperspectral images, the background should be removed. A binary image was formed using the gray-scale image at 1200 nm by setting the reflectance threshold value of 0.122. The sample region had the value of 1 and the background had the value of 0. A morphological processing method (opening operation) was conducted on the binary image to further reduce noise. Then the binary image was applied to gray-scale images at each wavelength to remove the background.

After the removal of background, the entire sample region of each chestnut was defined as a ROI, and pixel-wise spectra within the ROI were extracted. Due to the obvious random noises at the beginning and the end of spectra, only the spectral range of 975–1646 nm was studied. The pixel-wise spectra should be preprocessed to minimize the random noise before analyses. Taking the edge effect into account, we substituted a constant (0) for the edge of ROIs. Wavelet transformation using the wavelet function Daubechies 7 with decomposition level 3 was used to reduce the random noises [28,29]. After pixel-wise spectra preprocessing, pixel-wise spectra within each chestnut were then averaged as one spectrum to represent the sample.

### 2.5. Chemometric Methods

#### 2.5.1. Optimal Wavelength Selection

The Successive Projection Algorithm (SPA) is a forward-loop variable selection method that can extract valid information from the redundant spectral information, minimize the collinearity between spectral variables and improve the modeling conditions of multiple linear regression (MLR) model. SPA was applied to reduce the dimensionality of data in spectral domain of chestnuts during the study [30].

#### 2.5.2. Back Propagation Neural Network

The back propagation neural network (BPNN) is a multi-layer feed-forward artificial neural network with back-propagation algorithm. BPNN has the capability of large-scale parallel distributed processing with distributed information storage method. BPNN also has a strong self-adaptability to the environment and the learning ability of the outside world, as well as strong robustness and fault tolerance. BPNN is usually composed of an input layer, hidden layer and output layer. The layers are connected to each other, but nodes in each layer are not connected between each other. The number of its input layer neurons usually takes the value of the dimension of the input vector. And the number of output neurons usually takes the value of the dimension of the output vector. There is no definite standard for choosing the number of hidden layer neurons, so it is often decided by traversing within the set range [7,31,32].

**Figure 3:**
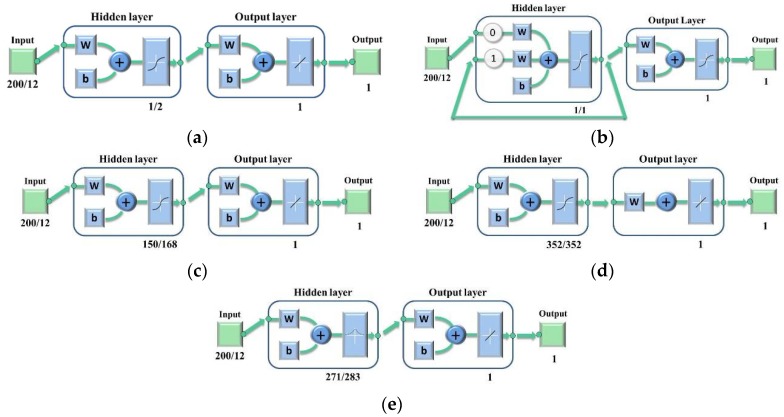
The topology structure of (**a**) BPNN models; (**b**) ENN models; (**c**) ELM models; (**d**) GRNN models; (**e**) RBNN models.

The topology structure of BPNN model is shown in Figure 3a. The input number was 200/12 for BPNN models using full spectra or optimal wavelengths (the former number was for full spectra and the later number was for optimal wavelength), respectively. The corresponding number of neurons of hidden layer was set as 1/2 according to the results of parameter optimization. The transfer functions of the hidden layer and output layer are the hyperbolic tangent sigmoid transfer function (‘tansig’) and linear transfer function (‘purelin’). ‘Tranlm’, a network training function that updates weight and bias values according to Levenberg-Marquardt optimization, is the BPNN training function.

#### 2.5.3. Evolutionary Neural Network

The evolutionary neural network (ENN) is a new neural network model based on two intelligent branches, the evolutionary computation and the neural network. One of the main features of the ENN model is its adaptability to dynamic environments. This adaptive process is realized by three levels of evolution, including the evolution of connection weights, network structures and learning rules [33]. Figure 3b reveals the topology structure of the ENN model. The transfer functions of hidden layer and output layer are both ‘tansig’. The training function of ENN is the same as BPNN. The number of neurons of hidden layer was also chosen according to parameter optimization.

#### 2.5.4. Extreme Learning Machine

The extreme learning machine (ELM) is a kind of single-hidden layer feed-forward neural network (SLFNN). Only the output weights are required to get the global optimal solution. ELM has fast learning ability, strong generalization ability and produces the only optimal solution. ELM algorithm can be used for discriminant analysis or regression analysis. In the practical application the number of neurons in the hidden layer needs to be determined, and it is currently determined by updating the parameter value to get optimum solution [25,34,35].

The topology structure of the ELM model is also shown in Figure 3c. Different from the BPNN model, the transfer functions of ELM are symbolic sine functions. Like BPNN and ENN, the only parameter that needs to be set for ELM is also the number of neurons in the hidden layer.

#### 2.5.5. General Regression Neural Network

The general regression neural network (GRNN) is a kind of radial basis function (RBF) network developed by Specht in 1991. It is a powerful regression tool with a dynamic network structure, whose training speed is extraordinarily fast. Its simplicity of the network structure and the implementation endows GRNN popularity in a variety of fields like image processing [36,37].

Figure 3d showed the topology of GRNN. The transfer function of GRNN is Levenberg-Marquardt backpropagation. Different from BPNN, ELM and ENN, the parameter to be optimized for GRNN is the spread value of its radial basis function. For each spread value, the Matlab program will automatically choose the best neurons, because of which the spread value is the only factor related to classification results that needs to be determined.

#### 2.5.6. Radial Basis Neural Networks

The radial basis neural network (RBNN) originates from the usage of radial basis functions in the solution of real multivariate interpolation problems. As a multilayer perceptron (MLP) they can approximate any regular function. Because of its local behavior and the linear nature of its output layer, it can be trained faster than other MLPs, which makes it useful in various applications. However, the generalization capability of RBNN is normally poor because it focuses too much on the training data set. Hidden-layer neurons represent regions of the input space, which makes the search of the appropriate number of hidden-layer neurons become a difficult task [38,39]. Spread value is also the only parameter needed to be optimized for RBNN. Different from BPNN, the most common transfer function of RBNN is the Gaussian function.

### 2.6. Principal Component Analsis

To explore qualitatively discrimination of healthy and moldy chestnuts, principal component analysis (PCA) was applied. PCA was conducted in two ways in this study, including PCA scores image and PCA scores scatter plots. PCA scores image were obtained by applying PCA on the hyperspectral images. The background was firstly removed from the hyperspectral images using the method described in Section 2.4. Then pixel-wise scores of each component were obtained to form the PCA scores image. The PCA scores image were used to explore qualitative pixel-wise differences between healthy and moldy chestnuts. Besides the PCA scores image, the scores scatter plots using scores from different principal components obtained by the sample average spectra were also used to explore the qualitative classification of healthy and moldy chestnuts.

## 3. Results and Discussion

### 3.1. Spectral Profiles

**Figure 4:**
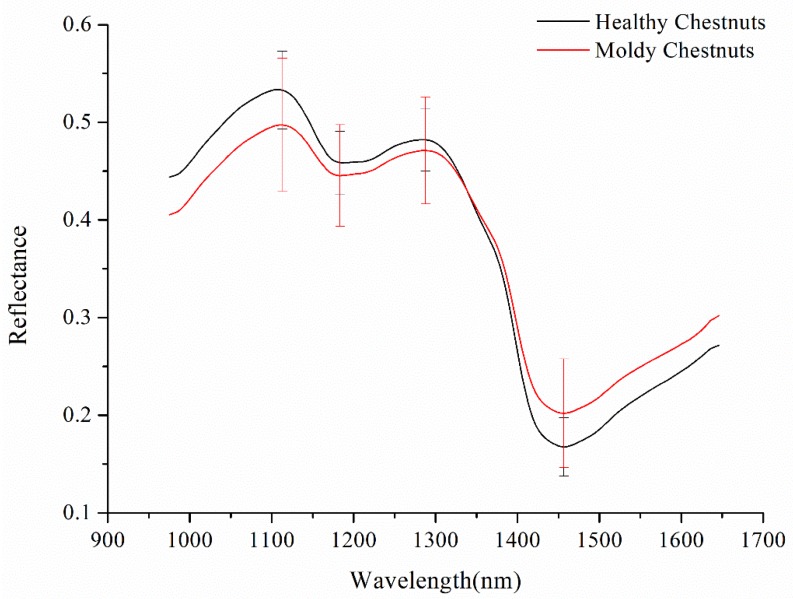
Average spectra with SD of healthy chestnuts and moldy chestnuts.

The spectral data in the 975–1646 nm range with 200 wavelength variables was studied. The average spectra of healthy chestnuts and moldy chestnuts with standard deviation (SD) of wavelengths at peaks and valleys are shown in Figure 4. It’s obvious that there was a large proportion of overlap between the two curves, so further study should be conducted in order to better distinguish between healthy chestnuts and moldy chestnuts.

### 3.2. PCA Scores Image Visualization

**Figure 5:**
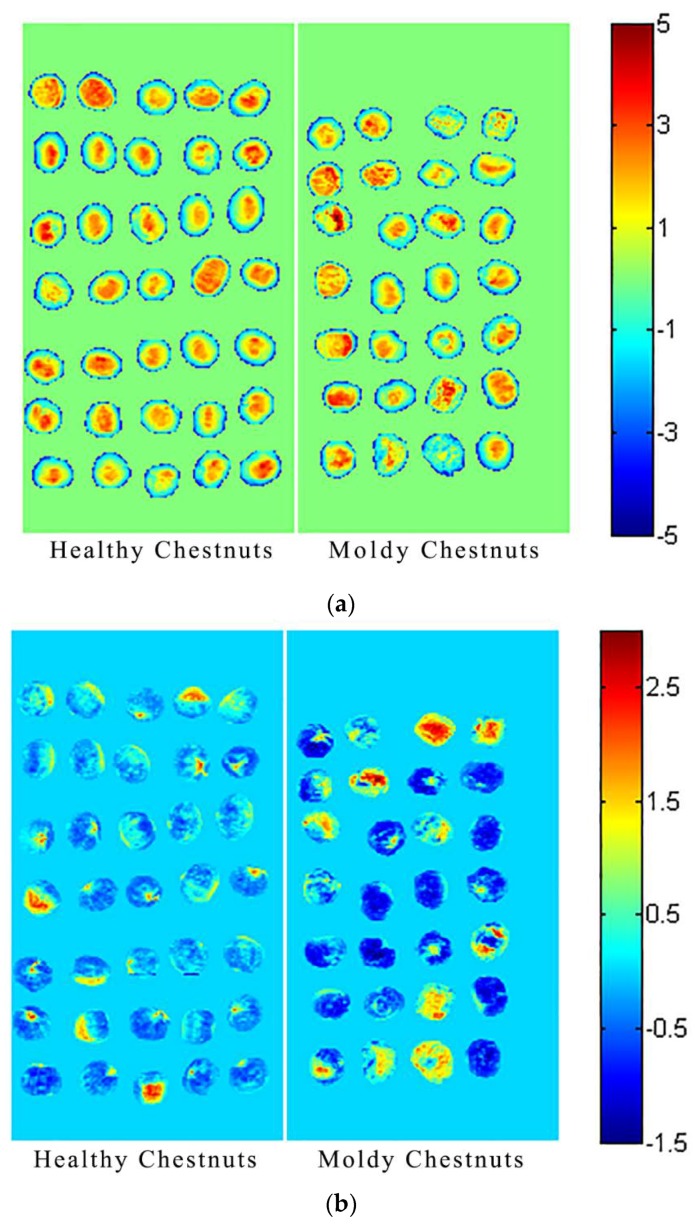
Scores image of chestnuts. (**a**) Healthy chestnuts and moldy chestnuts for the first principal component; (**b**) healthy chestnuts and moldy chestnuts for the second principal component.

PCA scores image could be depicted according to pixel-wise PCA scores. Figure 5a shows the visualized hyperspectral images for the first principal component (PC1) of healthy chestnuts and moldy chestnuts while Figure 5b shows the visualized hyperspectral images for the second principal component (PC2) of healthy chestnuts and moldy chestnuts.

As seen from Figure 5a, PC1 explained the most of the variance. All the images of chestnut samples showed a blue periphery (representing the edge of the chestnut) and an orange or yellow kernel (representing the top of the chestnut). The differences between the edge and the top might be attributed to the chestnut’s morphological information. It could also be found that there were no obvious differences between healthy chestnuts and moldy chestnuts, the reason might be that PC1 also contained the basic composition information of the chestnuts. The first principal component scores image might reveal the basic common information of chestnut samples, including morphological information and basic composition information.

It can be seen from Figure 5b, the proportion of blue area of moldy chestnut samples was darker than for healthy chestnut samples. Some of the chestnut samples showed yellow, orange and red colors, which represented the inside part of chestnut samples. As shown in Figure 5, healthy and moldy chestnuts had the potential to be distinguished from each other. It could also be found that the differences between edge and the top of chestnuts were not obvious. The reason might be that PC2 scores image mainly contained information of the differences between healthy and moldy chestnuts rather than morphological information.

### 3.3. PCA Scores Scattter Plot Analysis

**Figure 6:**
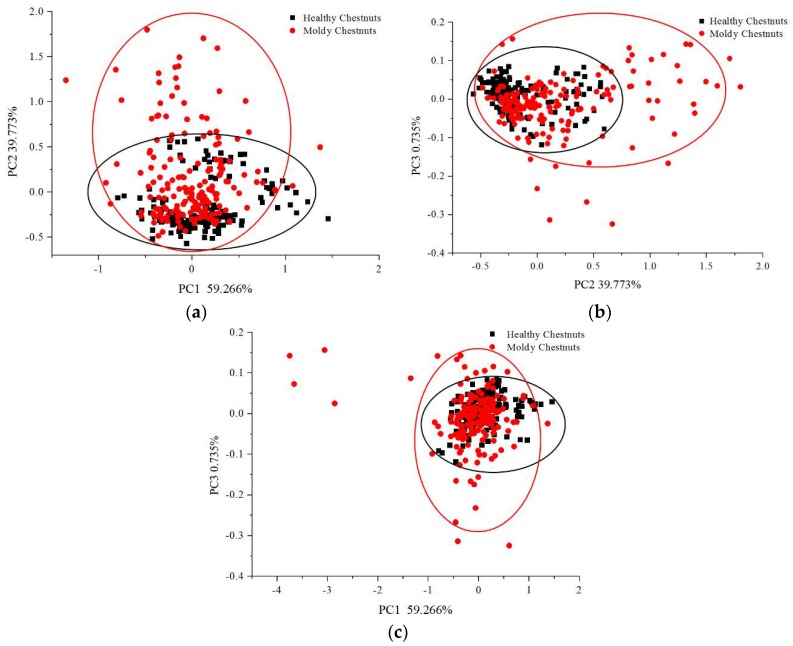
Score scatter plot of chestnuts. (**a**) PC1 vs. PC2; (**b**) PC2 vs. PC3; (**c**) PC1 vs. PC3.

PCA was performed on the average spectra data for qualitative discriminative analysis of healthy chestnuts and moldy chestnuts. The first three PCs explained over 99% of the total variance. Figure 6 shows the scores scatter plots of PC1 vs. PC2, PC2 vs. PC3 and PC1 vs. PC3.

To represent the distribution of chestnuts in Figure 6 more intuitively, ellipses were drawn to show the region covering the most healthy or infected chestnuts. As seen from Figure 6, both healthy chestnuts and moldy chestnuts gathered to their own cluster center, although there was some overlap between the two clusters. The points of healthy chestnuts were more concentrated while the points of moldy chestnuts were more scattered. The results indicated the potential of discriminating moldy chestnuts using hyperspectral imaging. Discrimination models should be further developed in order to obtain satisfactory healthy and moldy chestnut classification results.

### 3.4. Optimal Wavelengths Selection

The spectral data obtained by hyperspectral imaging systems usually have a large data volume and contain a lot of irrelevant information like redundant, collinear and background information. The existence of irrelevant information will not only affect the extraction of useful information, but also increase the data processing burden, which is likely to cause instability in the model and thus a poor performance. Meanwhile, the large amount of computation places a high requirement on computer hardware. The characteristic wavelengths are the wavelengths containing the useful information selected from a large number of wavelengths by removing the redundant and collinear data and wavelengths representing the background signal. The selection of characteristic wavelengths can reduce the inputs, simplify the model and improve the model performance.

In this study, SPA was adopted to select the optimal wavelengths. It can be seen from Table 1, 12 optimal wavelengths were selected from among 200 wavelengths to reduce the data volume.

### 3.5. Classification Models on Full Spectra and Optimal Wavelengths

In order to evaluate whether the infected chestnuts could be detected by hyperspectral imaging, BPNN, ENN, ELM, GRNN and RBNN models were built for comparison. There were 264 healthy chestnuts and 264 blue mold infected chestnuts. Each variety was split into a calibration set and a prediction set at a ratio of 2:1.

**Figure 7:**
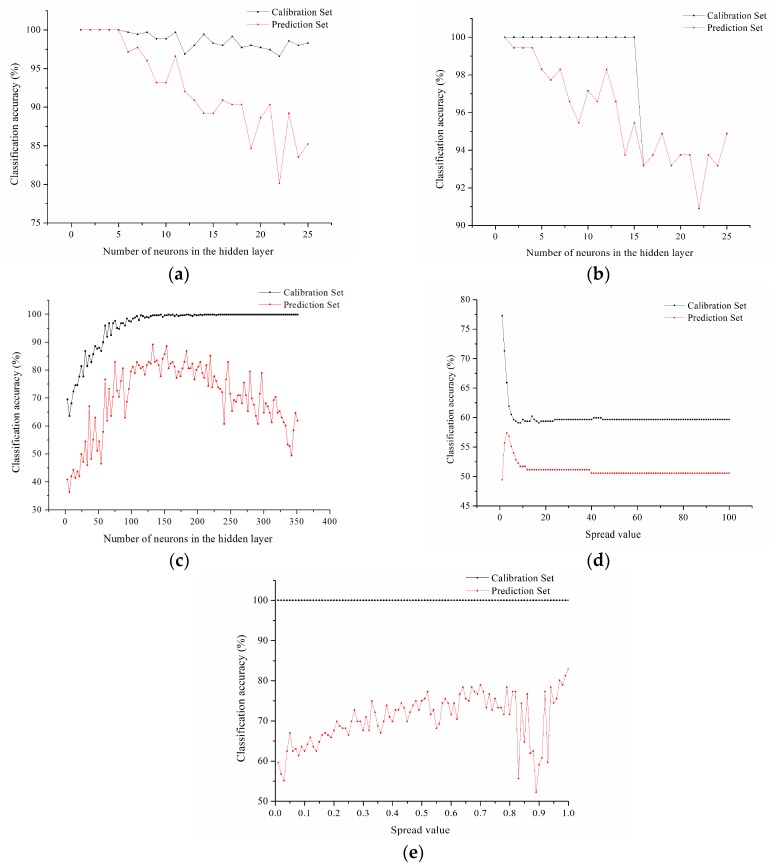
Parameter optimization of classification models using full spectra: (**a**) BPNN model; (**b**) ENN model; (**c**) ELM model; (**d**) GRNN model; (**e**) RBNN model.

In order to obtain a better classification results, the parameters of BPNN, ENN, ELM, GRNN and RBNN, which were the number of neurons (BPNN, ENN and ELM) or spread value (GRNN and RBNN) of the hidden layer, should be optimized first. The parameters were selected according to the classification accuracies of the calibration set and prediction set of each model. Figure 7 showed the changing trend of classification accuracy of calibration set with different number of neurons or spread value in the hidden layer of full spectra for five models. The more the number of neurons in the hidden layer, the slower the data processing speed was. Therefore, considering the condition of the highest accuracy of the calibration set, the minimum number of neurons should be chosen. According to the curve, the number of neurons in the hidden layer or spread value was chosen as 1, 1, 150, 2, 1 for BPNN, ENN, ELM, GRNN and RBNN models, respectively.

**Figure 8:**
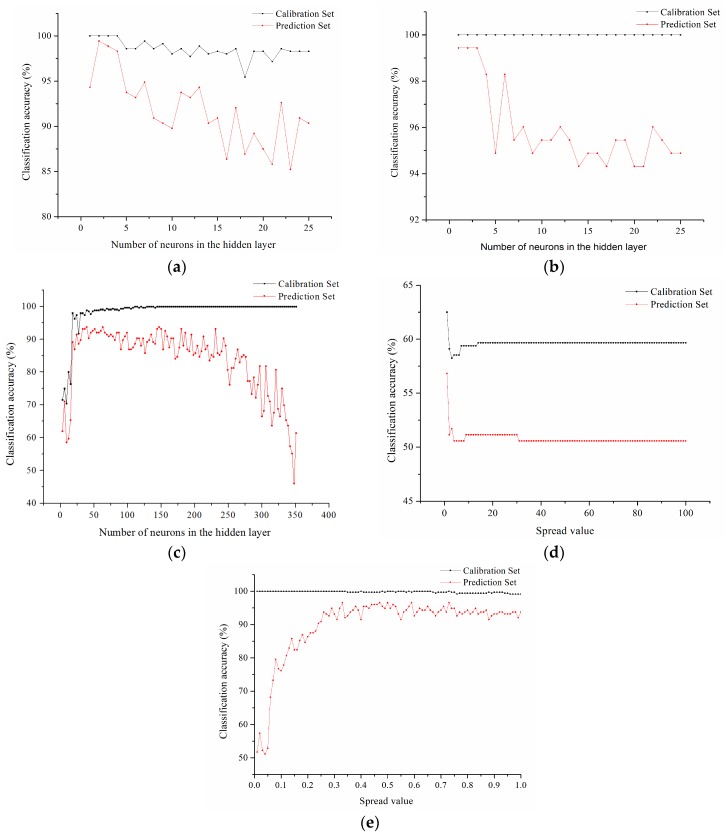
Parameter optimization of classification models based on optimal wavelengths: (**a**) BPNN model; (**b**) ENN model; (**c**) ELM model; (**d**) GRNN model; (**e**) RBNN model.

The changing trend of classification accuracy of calibration set with different parameters of classification models based on optimal wavelengths is shown in Figure 8. In order to get the best classification results as well as the fastest processing speed, 2, 1, 168, 1, 0.33 neurons or spread value were selected to be the parameters of the BPNN, ENN, ELM, GRNN and RBNN models using the optimal wavelengths, respectively.

Table 2 shows the result of the BPNN, ENN, ELM, GRNN and RBNN models using full spectra or optimal wavelengths. As shown in Table 2, most of the models using full spectra and optimal wavelengths obtained acceptable results.

For models using full spectra, the accuracy of the calibration set and the prediction set of BPN and ENN models all reached 100%. The accuracy of the calibration set of ELM and RBNN models was the same as for the BPNN and ENN models, reaching 100%. However, the accuracy of the prediction set of ELM and RBNN models was slightly lower than that of the BPNN and ENN models, which only surpassed 80%. The accuracy of the calibration set and the prediction set of GRNN model based on full spectra was much lower than that of other models, with 71.31% accuracy for the calibration set and 55.68% accuracy for the prediction set. 

For the models using optimal wavelengths, the accuracy of the calibration set of BPNN, ENN, ELM and RBNN models all reached 100%. However, the prediction accuracy of ELM model only reached 81.25%, while the prediction accuracy of three other models (BPNN, ENN and RBNN) all surpassed 96%. The BPNN, ENN and RBNN models performed the best among the five models. The prediction result of the ELM model based on optimal wavelengths was inferior to that of the BPNN, ENN and RBNN models with a prediction accuracy of 81.25%. The GRNN model based on optimal wavelengths showed the worst results among the five models, with 62.5% accuracy for the calibration set and 56.82% accuracy for the prediction set. 

The selection of optimal wavelengths reduced the volume of data tremendously, and improved the speed of modeling and prediction, especially for the BPNN, ENN and RBNN models. Comparing the classification results of the models based on full spectra with the models using optimal wavelengths in Table 2, similar results could be found. The accuracy of the prediction set of the BPNN and ENN models using full spectra and optimal wavelengths all reached 100%. Although the classification results of the prediction set based on optimal wavelengths were slightly lower than those based on full spectra, the prediction accuracy was still higher than 99%. The results of the BPNN and ENN models were more stable than those of the other models. As for the ELM models, the accuracy of the prediction set based on optimal wavelengths was inferior to that based on full spectra. As for the GRNN models, the accuracy of the calibration set based on optimal wavelengths fell by about 10%, while the accuracy of the prediction set just had a small fluctuation. Generally, the GRNN model based on optimal wavelengths or full spectra performed poorly compared with the other four models. The poor performance of the GRNN model might be due to the fact that its topology structure, transfer function or training function weren’t applicable for moldy chestnut detection. In contrast with the models based on full spectra, the RBNN model based on optimal wavelengths showed better result in the calibration set, with accuracy raising up to 96.59%. RBNN was the only one among the five models which performed better as a model based on optimal wavelengths than a model using full spectra.

## 4. Conclusions

A new method for discrimination of moldy chestnuts was proposed in this study. A hyperspectral imaging system with a wavelength range of 874–1734 nm was applied to identify moldy chestnuts. PCA score visualization images were depicted to distinctly show the differences between healthy and moldy chestnuts. In order to reduce the training time of models, 12 optimal wavelengths were selected by SPA. Artificial neural networks such as BPNN, ENN, ELM, GRNN and RBNN were used to build models based on full spectra and optimal wavelengths. Parameter optimization was conducted in the five models in order to improve the classification accuracy. All models except for the GRNN model achieved satisfactory results. Among all the models using full spectra and optimal wavelengths, the BPNN and ENN models obtained the highest accuracy with all classification accuracies reaching above 99%. The results showed the feasibility of using hyperspectral imaging combined with an optimal wavelengths selection method and artificial neural networks to discriminate moldy chestnuts.

Further studies are expected to conduct more research on how to distinguish moldy chestnuts with different varieties of mold species using hyperspectral imaging system. More chestnut samples from different regions should be taken into account in order to improve the robustness of models. Besides, more study should be conducted on how to improve the universality of the discrimination models.

## Figures and Tables

**Table 1:** The optimal wavelengths selected by SPA.

Number	Optimal Wavelengths (nm)
12	1005, 1012, 1116, 1156, 1305, 1332, 1392, 1399, 1517, 1592, 1622, 1646

**Table 2:** BPNN, ENN, ELM, GRNN and RBNN models using full spectra or optimal wavelengths.

Classification Model	Full Spectra				Optimal Wavelengths			
	Parameter	Cal ^a^ (%)	Pre ^b^ (%)	Com ^c^ (s)	Parameter	Cal (%)	Pre (%)	Com (s)
BPNN	1 ^d^	100	100	893.51	2	100	99.43	37.76
ENN	1 ^d^	100	100	33,607.31	1	100	99.43	614.80
ELM	150 ^d^	100	87.50	6.28	168	100	81.25	5.07
GRNN	2 ^e^	71.31	55.68	10.55	1	62.50	56.82	9.08
RBNN	1 ^e^	100	82.96	952.21	0.33	100	96.59	375.16

^a^ The accuracy of the calibration set; ^b^ the accuracy of the prediction set; ^c^ computation time; ^d^ number of neurons in the hidden layer; ^e^ spread value of the radial basis function.
